# Gesundheit und Gesundheitsversorgung von trans Personen während der COVID‑19-Pandemie: Eine Online-Querschnittstudie in deutschsprachigen Ländern

**DOI:** 10.1007/s00103-021-03432-8

**Published:** 2021-10-07

**Authors:** Daria Szücs, Andreas Köhler, Mika M. Holthaus, Annette Güldenring, Lena Balk, Joz Motmans, Timo O. Nieder

**Affiliations:** 1 Institut für Sexualforschung, Sexualmedizin und Forensische Psychiatrie, Interdisziplinäres Transgender Versorgungscentrum Hamburg, Universitätsklinikum Hamburg-Eppendorf, Martinistraße 52, 20246 Hamburg, Deutschland; 2 Klinik für Psychiatrie, Psychotherapie und Psychosomatik, Westküstenklinikum Heide, Heide, Deutschland; 3 BVT* e. V. (Bundesverband Trans* e. V.), Schiffbauerdamm 8, 10117 Berlin, Deutschland; 4 dgti e. V. (Deutsche Gesellschaft für Transidentität und Intersexualität e. V.), Braunschweig, Deutschland; 5 Transgender Infopunt, Ghent University Hospital, Gent, Belgien

**Keywords:** Transgender, Geschlechtsdysphorie, Trans-Gesundheit, COVID‑19, Psychische Belastung, Transgender, Gender dysphoria, Transgender health, COVID‑19, Mental health

## Abstract

**Einleitung und Ziel:**

Seit dem Frühjahr 2020 hat die COVID‑19-Pandemie nahezu alle Bereiche des gesellschaftlichen Lebens erheblich eingeschränkt, was bei vielen Menschen sowohl zu psychischen als auch zu körperlichen Belastungen geführt hat. In diesem Artikel nehmen wir die Situation von trans Personen in den Blick, die infolge ihrer gesellschaftlichen Diskriminierung und Marginalisierung sowie spezifischer, gesundheitsbezogener Anliegen durch eine besondere Vulnerabilität gekennzeichnet sein können.

**Methoden:**

Unter Beachtung partizipativer Elemente haben wir mit einer Online-Querschnitterhebung im Zeitraum vom 01.05.2020 bis zum 31.01.2021 die psychische und physische Gesundheit von trans Personen im deutschsprachigen Raum sowie deren Zugang zur Trans-Gesundheitsversorgung während der COVID‑19-Pandemie untersucht.

**Ergebnisse:**

Trans Personen erleben seit Beginn der COVID‑19-Pandemie vermehrt Barrieren sowohl bei geschlechtsangleichenden Behandlungen und psychosozialen Unterstützungsangeboten als auch im Bereich der COVID‑19-bezogenen medizinischen Versorgung. Im Vergleich zur Gesamtbevölkerung berichten sie übermäßig häufig von somatischen Erkrankungen, auch von solchen, die ein erhöhtes Risiko für schwere Verläufe einer COVID‑19-Infektion darstellen. Außerdem berichten die Teilnehmenden verschiedene Faktoren, die ein Risiko für eine erhöhte psychische Belastung darstellen können (z. B. Zugehörigkeit zu einer Minderheit aufgrund nicht-heterosexueller Orientierung, niedriges Einkommen).

**Diskussion:**

Die Ergebnisse unserer Untersuchung weisen darauf hin, dass bisherige Vulnerabilitäten für gesundheitliche Probleme und der eingeschränkte Zugang zu einer qualifiziert informierten Trans-Gesundheitsversorgung durch die Folgen der COVID‑19-Pandemie verschärft wurden.

## Abstract (en)

**Background and objective:**

Since spring of 2020, the COVID‑19 pandemic has disrupted our day-to-day lives and led to negative consequences in various areas of life, including mental and physical wellbeing. In this article, we take a closer look at the situation of trans people, who – due to experiences with discrimination and marginalization as well as their specific health-related interests – could be characterized by a particular vulnerability.

**Methods:**

Using an online cross-sectional survey, which we designed collaboratively with experts from the trans community, we investigated the mental and physical health of trans people from German-speaking countries and their access to trans-related healthcare during the COVID‑19 pandemic in the period from 1 May 2020 to 31 January 2021.

**Results:**

Since the beginning of the COVID‑19 pandemic, trans people have experienced barriers in access to gender-affirming treatments, mental health services, and COVID‑19-related medical care. At the same time, trans people reported being affected by chronic diseases disproportionately more often than the general population, including those leading to a higher risk for poorer outcomes of a COVID‑19 infection. Moreover, the participants reported being exposed to many risk factors associated with higher mental distress (e.g., having a chronic illness, belonging to a minority based on a non-heterosexual orientation, or having a low income).

**Discussion:**

The results of this survey indicate that prior vulnerabilities with regards to health problems and the restricted access to an informed and qualified transgender healthcare were exacerbated by the COVID‑19 pandemic.

## Einleitung

Während der COVID‑19-Pandemie stehen epidemiologische, virologische und pathologische Fragestellungen weltweit im Zentrum der Gesundheitsforschung und gesundheitspolitischer Debatten. Die pandemiebedingten Auswirkungen auf die psychische Gesundheit werden im Zuge dessen häufig vernachlässigt und die Bedeutung psychologischer Aspekte für die Gesamtdynamik der COVID‑19-Pandemie unterschätzt [1, 2]. Dabei ist es im Verlauf der COVID‑19-Pandemie zu einem weltweiten Anstieg von psychischen Belastungen gekommen [3]. So wurde in Deutschland, der Schweiz und Österreich während des ersten *Lockdowns *eine Zunahme an Depressions- und Angstsymptomen verbunden mit subjektiv erlebter Einsamkeit und einer Abnahme der Lebensqualität beobachtet [4–7]. Sowohl Personen, die bereits an einer psychischen Störung litten [8, 9] als auch jene mit körperlichen Vorerkrankungen, die mit einem erhöhten Infektionsrisiko für COVID‑19 verbunden waren, waren von den psychischen Folgen stärker betroffen [9]. Es kam hinzu, dass aufgrund der Kontaktbeschränkungen der Zugang zu psychotherapeutischen und somatischen Behandlungen verzögert oder erschwert wurde [10]. Daher sollten psychosoziale Aspekte als Co-Faktoren bei der Pandemiebekämpfung beachtet werden. Dies gilt insbesondere für Menschen, die einem Minoritätenstress^1^ ausgesetzt sind, der sie auch ohne Pandemiefolgen anfällig für die Entwicklung psychischer Störungen macht.

Vor diesem Hintergrund richtet der vorliegende Beitrag den Fokus auf die Lebenssituation von trans Menschen und ihre Gesundheitsversorgung und fragt, ob und inwiefern diese von den Folgen der COVID‑19-Pandemie besonders betroffen sein könnten [11–16]. Das Wort „trans“ verwenden wir im vorliegenden Beitrag als Sammelbegriff für Menschen, die sich nicht bzw. nicht vollständig mit dem ihnen bei Geburt zugewiesenen Geschlecht identifizieren. Der Begriff dient als Kurzform für unterschiedliche Beschreibungen einer individuellen Geschlechtlichkeit (z. B. transgender, transident, transsexuell; [17]). Die geschlechtliche Selbstbezeichnung variiert bei trans Personen zwischen binär (männlich oder weiblich) und non-binär (weder ausschließlich männlich noch weiblich). Das kann beispielsweise bedeuten, dass sich eine Person als oszillierend zwischen verschiedenen Geschlechtern erlebt (z. B. genderfluid) oder eine Geschlechtszuschreibung als Ganzes ablehnt (z. B. agender).

Klinisch bedeutsam sind geschlechtlich variante Lebensformen dann, wenn sie die diagnostischen Kriterien einer Geschlechtsinkongruenz oder Geschlechtsdysphorie erfüllen. Als Diagnose wurde die Geschlechtsdysphorie (GD) von der *American Psychiatric Association* (APA) im DSM‑5, der fünften Auflage des Diagnostic and Statistical Manual of Mental Disorders, eingeführt, die den Leidensdruck aufgrund einer ausgeprägten Diskrepanz zwischen geschlechtlichem Erleben und Zuweisungsgeschlecht beschreibt [18]. Die 11. Version der internationalen statistischen Klassifikation der Krankheiten und verwandter Gesundheitsprobleme (ICD-11) spricht von Geschlechtsinkongruenz (GI; [19]). Die Inhalte einer transitionsunterstützenden^2^ Behandlung verfolgen ein individuelles Konzept, in dem sowohl begleitende psychotherapeutische Ansätze als auch somatische Eingriffe (hormonell, chirurgisch) im Zuge einer Geschlechtsangleichung indiziert sein können [17].

Als marginalisierte Gruppe erfahren trans Personen im Vergleich zur Cis^3^-Bevölkerung im sozialen Raum überzufällig häufig Diskriminierungen, z. B. in Form von Benachteiligung bei der Arbeits- und Wohnungssuche [20], und leiden unter körperlicher und sexueller Gewalt [20, 21]. Neben der konkreten Belastung durch Benachteiligung und Gewalt kann der resultierende Minoritätenstress die Vulnerabilität für psychische und körperliche Erkrankungen noch verstärken. Doch auch auf der Suche nach medizinischer oder psychotherapeutischer Behandlung erleben trans Personen strukturelle Diskriminierung und Barrieren, die von der Ansprache mit einer geschlechtsbezogenen falschen Anrede (das sog. misgendern) bis zur Behandlungsverweigerung reichen [23]. Das resultiert vor allem daraus, dass Behandler:innen^4^ häufig unzureichend über transbezogene Probleme und Versorgungsbedarfe informiert sind [27]. Hier zu berücksichtigen ist, dass trans Personen, die Diskriminierung im und durch das Gesundheitssystem erleben, selbiges tendenziell meiden [23, 27]. Dies ist insofern besorgniserregend, weil diese Vermeidung auch im Fall einer körperlichen oder psychiatrischen Erkrankung wirksam ist [27]. Im Zusammenhang mit geschlechtsangleichenden somatischen Behandlungen erleben trans Personen zudem im Bezug auf die Kostenübernahme medizinischer Maßnahmen erhebliche Barrieren. Sie sind für die Kostenübernahme von geschlechtsangleichenden Behandlungen auf eine Diagnose- und Indikationsstellung durch approbierte Psychotherapeut:innen oder Psychiater:innen angewiesen [28], die wiederum sozialmedizinisch in jedem Einzelfall geprüft wird [29]. Zudem sind qualifizierte und informierte Fachkräfte selten und praktizieren eher in Großstädten [30]. Diese defizitäre Situation führt für trans Personen zu einer begrenzten Auswahlmöglichkeit zwischen geeigneten Behandler:innen, langen Wartezeiten bis zu einem Erstkontakt sowie zu zeit- und kostenintensiven Anfahrten zum Behandlungsort [30].

### Einschränkungen während der COVID‑19-Pandemie

Es gibt bereits Hinweise darauf, dass bestehende Vulnerabilitäten von trans Personen durch die pandemiebedingten Einschränkungen verschärft wurden. So berichtet eine internationale Untersuchung, dass weltweit der Zugang zu transitionsunterstützender Versorgung seit Beginn der COVID‑19-Pandemie eingeschränkt wurde [11]. Beratung und Therapie für die Behandlung psychischer Störungen waren am häufigsten von den Einschränkungen betroffen, es folgten ein eingeschränkter Zugang zur Hormontherapie und zur Nachsorge nach Operationen. Fachkräfte aus einer auf Transgender-Versorgung spezialisierten Klinik in Peking, China, berichten ebenfalls, dass der Zugang zu Hormonbehandlungen eingeschränkt und Operationen verschoben wurden [13]. Auch die Nachsorge im Anschluss an operative Eingriffe habe nicht stattfinden können [13]. In den Niederlanden wurde außerdem über vermehrte Barrieren zur allgemeinen Versorgung berichtet [14]. Soziale Isolierung und ein erschwerter Zugang zu psychosozialer (z. B. Sozialarbeit und communitybasierte Hilfen) sowie psychotherapeutischer Unterstützung (z. B. fehlende Angebote für eine telemedizinische Behandlung) könnten das Risiko für psychische Störungen erhöhen [14]. Trans Personen in den USA berichteten neben den zunehmenden Barrieren zur Trans-Gesundheitsversorgung und zunehmender psychischer Belastung außerdem, dass die wahrgenommene Unterstützung durch die Trans-Community abgenommen habe [12].

Zusammenfassend zeigt sich, dass die Gesundheit von trans Personen während der COVID‑19-Pandemie übermäßig gefährdet sein könnte. In der vorliegenden Arbeit berichten wir Daten zu dieser Fragestellung aus deutschsprachigen Ländern. Die Studie fokussiert hierbei auf die psychische und physische Gesundheit von trans Personen in Deutschland, der Schweiz und Österreich sowie auf Einschränkungen im Zugang zur Trans-Gesundheitsversorgung seit Beginn der COVID‑19-Pandemie.

## Methoden

### Studiendesign

Die zugrunde liegenden Daten stammen aus einem internationalen Online-Survey zur Situation der Trans-Gesundheitsversorgung während der COVID‑19-Pandemie:* TransCareCovid-19* (www.transcarecovid-19.com). Die TransCareCovid-19-Studie ist eine Querschnittstudie, die von Andreas Köhler, Joz Motmans und Timo O. Nieder erarbeitet und weltweit in Kooperation mit 23 Communityorganisationen sowie Fachkräften weiterentwickelt wurde. Der Survey wurde in 27 Sprachen übersetzt. In die Konzeption der deutschen Version des Survey waren neben den Wissenschaftler:innen des Universitätsklinikums Hamburg-Eppendorf (UKE) und des Westküstenklinikums Heide, der Bundesverband Trans* (BVT*), die deutsche Gesellschaft für Transidentität und Intersexualität (dgti e. V.) und Transgender Europe (TGEU) involviert. Am gesamten Forschungsprozess war eine Vielzahl von Personen beteiligt, die sich als Angehörige der LGBTQ+-Community^5^ verstehen. Die Entwicklung, Übersetzung und Distribution des Survey wurden partizipativ mit den beteiligten Autor:innen erarbeitet und hatten zum Ziel, die Wirklichkeit der Zielgruppe möglichst lebensweltnah zu erfassen. Obgleich sich ein partizipatives Vorgehen für die Forschung mit marginalisierten Populationen, wie z. B. trans Personen [31], besonders empfiehlt, kann die Studie dem Anspruch, durchgehend partizipativ gearbeitet zu haben, nicht gerecht werden.

### Stichprobe

Zur Teilnahme an der Studie eingeladen wurden Personen, die sich als trans identifizieren und zum Zeitpunkt der Erhebung mindestens 16 Jahre alt waren. Rekrutiert wurde über LGBTQ+-relevante soziale Medien, Mailinglisten von Trans-Verbänden und Organisationen sowie über ein Schneeballverfahren. Daten wurden seit Mai 2020 gesammelt. Der vorliegende Artikel berücksichtigt Daten aus den deutschsprachigen Ländern, die bis zum 31.01.2021 erhoben wurden.

### Maße

Der Fragebogen beinhaltete Items zu folgenden demografischen Daten: Alter, bei Geburt zugewiesenes Geschlecht, geschlechtliche Selbstbezeichnung, Bildungsgrad, Beschäftigungsstand, Wohnsitz (Land, städtische vs. ländliche Region), Einkommen und Minderheitenstatus (People of Color, religiöse Minderheit, sexuelle Minderheit, Minderheit wegen der geschlechtlichen Selbstbezeichnung, Selbstbezeichnung als Personen mit Behinderung, andere). Der Gesundheitsstatus wurde über Items aus vorherigen Studien [32] und Freitextantworten erhoben. Items, die die Erfahrungen mit COVID‑19 erfragten (z. B. COVID‑19-Symptomatik in den letzten 14 Tagen, Kontakte mit an COVID‑19 erkrankten Personen) orientierten sich ebenfalls an vorliegenden Studien [33]. Mit Bezug auf COVID‑19 wurden transspezifische Diskriminierungserfahrungen und die Vermeidung von medizinischer Versorgung erfragt. Weiterhin wurden Daten zur Inanspruchnahme von geschlechtsangleichenden Behandlungen und der Einfluss der COVID‑19-Pandemie auf den Zugang zu diesen Behandlungen erhoben. Auch der Zugang zur Unterstützung durch die Trans-Community – in Form von Selbsthilfegruppen oder Trans-Beratungsstellen – und der Zugang zur Psychotherapie wurden erhoben. Über eine Pfadabhängigkeit wurden den Teilnehmenden (TN) ausschließlich Fragen zu Behandlungen gestellt, die sie bereits in Anspruch genommen hatten. Zum Beispiel wurden nur TN, die schon eine Hormontherapie begonnen hatten, gefragt, ob der Zugang zu dieser eingeschränkt war. Zuletzt wurde die psychische Belastung der TN mit dem *Brief Symptom Inventory *(BSI-18) erhoben [34]. Der BSI-18 beinhaltet 18 Items, die psychische Belastung aus 3 Bereichen (Depressivität, Ängstlichkeit, Somatisierung) erfassen. Die Items werden auf einer Likert-Skala von „überhaupt nicht“ (0) bis zu „sehr stark“ (4) beantwortet. Diese werden zu einem Gesamtwert – dem *Global Severity Index *(globaler Schweregradindex, GSI) – zusammengefasst, der die allgemeine psychische Belastung widerspiegelt [34].

### Statistische Analysen

Demografische Daten, Gesundheitsstatus sowie die Einschränkungen der Trans-Gesundheitsversorgung wurden mittels deskriptiver Statistik analysiert. Für kontinuierliche Daten geben wir Mittelwerte und Standardabweichungen an, für kategoriale Daten werden die Häufigkeiten und Prozentanteile berichtet. Fehlende Daten wurden paarweise aus den Analysen ausgeschlossen. Aspekte, die zu vermehrten Einschränkungen im Zugang zu geschlechtsangleichenden Maßnahmen führten, haben wir mit einer multiplen logistischen Regression analysiert. Dabei haben wir folgende Prädiktoren untersucht: die geschlechtliche Selbstbezeichnung (binär/nichtbinär), das bei Geburt zugewiesene Geschlecht (männlich/weiblich), das monatliche Einkommen (als kontinuierliche Variable mit höheren Werten für ein niedrigeres Einkommen), die Zugehörigkeit zu einer religiösen Minderheit, einer sexuellen Minderheit, die Selbstbezeichnung als Person mit Behinderung und das Vorhandensein mindestens einer körperlichen Erkrankung. Die Voraussetzungen der logistischen Regression (Linearität des Logits, keine Multikollinearität) lagen vor. Wir haben keine Ausreißer mit einem überproportionalen Einfluss auf das Modell gefunden. Cox-Snells R^2^ und Nagelkerkes R^2^ werden als Indikatoren für die Modellanpassung angegeben. Zuletzt wurde die psychische Belastung mit einer multiplen linearen Regression untersucht, wobei der GSI als Kriterium diente. In diesem Modell untersuchten wir folgende Prädiktoren: die geschlechtliche Selbstbezeichnung (binär/nichtbinär), das bei Geburt zugewiesene Geschlecht (männlich/weiblich), das Einkommen (als kontinuierliche Variable mit höheren Werten repräsentativ für ein niedrigeres Einkommen), die Zugehörigkeit zu einer religiösen Minderheit, einer sexuellen Minderheit, die Selbstbezeichnung als Person mit Behinderung, das Vorhandensein mindestens einer körperlichen Erkrankung und der eingeschränkte Zugang zu mindestens einer geschlechtsangleichenden Behandlung. Wir haben die Voraussetzungen für eine multiple lineare Regression und das Vorkommen von Ausreißern mit den entsprechenden Diagnoseverfahren analysiert [35].

## Ergebnisse

### Demografische Daten

In dem Zeitraum vom 01.05.2020 bis zum 31.01.2021 haben 2125 Personen an der deutschen Version der Studie teilgenommen. Wir haben 618 TN ausschließen müssen, weil diese weniger als 50 % des Fragebogens beantwortet hatten. Insgesamt haben wir die Daten von *N* = 1507 Teilnehmenden analysieren können, die zu 77 % aus Deutschland stammten. Die TN waren im Mittel 33,06 Jahre alt (SD = 13,08 Jahre). 1106 (73,4 %) TN nutzten eine binäre geschlechtliche Selbstbezeichnung, 334 (22,2 %) eine nichtbinäre geschlechtliche Selbstbezeichnung. Weitere Informationen zu den demografischen Daten finden sich in Tab. 1.

**Table 1:** 

Variablen	Absoluter und relativer Anteil
Alter in Jahren (Mittelwert (Standardabweichung)): 33,06 (13,08)	
*Höchster Bildungsabschluss*	
Kein formeller Bildungsabschluss	23 (1,5 %)
Haupt‑/Realschulabschluss	183 (12,1 %)
Allgemeine Hochschulreife	380 (25,2 %)
Berufsausbildung	250 (16,6 %)
Hochschulausbildung	464 (30,8 %)
Anderer Abschluss	99 (6,6 %)
Ich kann oder möchte diese Frage nicht beantworten	38 (2,5 %)
*Beruflicher Status (Mehrfachauswahl möglich)*	
Schüler:in, Student:in	474 (31,5 %)
In der Ausbildung	104 (6,9 %)
Ungelernte:r Arbeiter:in	34 (2,3 %)
Angelernte:r Arbeiter:in	43 (2,9 %)
Angestellte:r	509 (33,8 %)
Beamt:in	31 (2,1 %)
Selbstständige:r	132 (8,8 %)
Arbeit im informellen Sektor (z. B. als haushaltsführende Person)	16 (1,1 %)
Arbeitslos/ohne bezahlte Arbeit	171 (11,3 %)
In Ruhestand/pensioniert	41 (2,7 %)
Arbeitsunfähig	108 (7,2 %)
Anderer Status	90 (6,0 %)
Ich kann oder möchte diese Frage nicht beantworten	9 (0,6 %)
*Minderheitsstatus* (Selbstbezeichnung; Mehrfachauswahl möglich)	
Person of Color	134 (9,0 %)
Religiöse Minderheit	166 (11,1 %)
Sexuelle Minderheit	1305 (86,6 %)
Geschlechtliche Minderheit	1246 (82,7 %)
Person mit Behinderung	320 (21,3 %)
Andere Minderheit	344 (22,8 %)
*Geschlechtliche Selbstbezeichnung*	
(Trans/transsexueller) Mann	618 (41,0 %)
(Trans/transsexuelle) Frau	488 (32,4 %)
Cross-Dresser	13 (0,9 %)
Non-binary/nichtbinär/genderqueer/agender/polygender/gender-fluid	334 (22,2 %)
Ich weiß nicht, ich habe keine Präferenz	44 (2,9 %)
Antwort fehlt	10 (0,7 %)
*Wie oft leben Sie zurzeit entsprechend Ihres Geschlechts?*	
Nie	45 (3,0 %)
Gelegentlich	146 (9,7 %)
Meistens	274 (18,2 %)
Immer	1034 (68,6 %)
Antwort fehlt	8 (0,5 %)
*Bei der Geburt zugewiesenes Geschlecht*	
Weiblich	874 (58,0 %)
Männlich	620 (41,1 %)
*Wo leben Sie gegenwärtig?*	
Deutschland	1161 (77,0 %)
Österreich	88 (5,8 %)
Schweiz	235 (15,6 %)
Anderes Land	23 (1,5 %)
In einer Großstadt, im Einzugsgebiet einer Großstadt oder in einer mittelgroßen Stadt	991 (65,8 %)
In einem Dorf oder einer Kleinstadt	516 (34,2 %)
Veränderung der Wohnsituation wegen COVID‑19	122 (8,1 %)
Belastung wegen der aktuellen Wohnsituation	455 (30,2 %)
*… Ihr gesamtes monatliches Haushaltseinkommen. Kommen Sie mit Ihrem Einkommen ...?*	
Sehr gut aus	241 (16,0 %)
Gut aus	344 (22,8 %)
Ziemlich problemlos aus	388 (25,7 %)
Mit einigen Schwierigkeiten aus	283 (18,8 %)
Mit Schwierigkeiten aus	114 (7,6 %)
Mit großen Schwierigkeiten aus	91 (6,0 %)
Ich möchte die Frage nicht beantworten	21 (1,4 %)
Ich weiß es nicht	23 (1,5 %)

### Aktuelle Gesundheitssituation und Erfahrungen mit COVID‑19

Mehr als die Hälfte der TN (*n* = 826; 54,8 %) gab an, mindestens an einer der erfragten körperlichen Erkrankungen zu leiden. Darüber hinaus ergab sich aus der Analyse der Freitextantworten zum Gesundheitsstatus, dass etwa ein Drittel der Befragten (*n* = 492, 32,6 %) an einer psychischen Störung litt. Aufgrund von Problemen, die nicht mit der geschlechtlichen Selbstbezeichnung assoziiert waren, befanden sich 565 (37,5 %) der TN in psychotherapeutischer Behandlung. Allerdings konnten 299 (53,1 %) der TN die Therapie seit Beginn der COVID‑19-Pandemie nur eingeschränkt weiterführen. Hinsichtlich der COVID‑19-Versorgung haben sich 41 (2,7 %) der TN seit Beginn der COVID‑19-Pandemie auf SARS-CoV‑2 testen lassen, wobei 31 TN (2,1 %) Diskriminierung und 30 TN (2,0 %) Fehlbehandlungen bei der Testung erlebt haben. Weitere Informationen zum Gesundheitsstatus finden sich in Tab. 2.

**Table 2:** 

Angaben der Teilnehmenden	Absoluter und relativer Anteil
*Körperliche Erkrankungen*	
Psychische Störungen	492 (32,6 %)
Rückenschmerzen	485 (32,2 %)
Knieschmerzen	254 (16,9 %)
Hormonelle Erkrankungen	159 (10,2 %)
Erkrankungen des Herz-Kreislauf-Systems	150 (10,0 %)
Asthma	130 (8,6 %)
Erkrankungen der oberen Atemwege	63 (4,2 %)
Neurologische Erkrankungen	45 (3,0 %)
Rheuma	39 (2,6 %)
Osteoporose/Osteopenie	31 (2,1 %)
Chronische obstruktive Lungenerkrankung	20 (1,3 %)
Bluterkrankungen	17 (1,1 %)
Lungenerkrankung	16 (1,1 %)
HIV	16 (1,1 %)
Glaukom	13 (0,9 %)
Katarakt	11 (0,7 %)
Infektionserkrankungen	10 (0,7 %)
Krebs	8 (0,5 %)
*Psychotherapeutische Behandlung*	
Inanspruchnahme von psychotherapeutischen Behandlungen (z. B. wegen einer Depression)	565 (37,5 %)
Eingeschränkter Zugang zu einer psychotherapeutischen Behandlung	299 (53,1 %^a^)
Sorgen, dass der Zugang zu psychotherapeutischer Behandlung wegen COVID‑19 in der Zukunft eingeschränkt wird	267 (48,0 %^a^)
*Erfahrungen mit COVID‑19*	
In den letzten 14 Tagen auf COVID‑19 getestet	41 (2,7 %)
In den letzten 14 Tagen in Quarantäne	216 (14,3 %)
Mit COVID‑19 diagnostiziert	2 (0,1 %)
COVID‑19 überstanden	1 (0,1 %)
Direkter Kontakt mit einer Person, die nachweislich an COVID‑19 erkrankt war oder ist	30 (2,0 %)
Indirekter Kontakt mit einer Person, die nachweislich an COVID‑19 erkrankt war oder ist	73 (4,8 %)
Kontakt mit einer Person, bei der es einen Verdacht auf COVID‑19 gab oder gibt	301 (20,0 %)
*COVID‑19-bezogene Gesundheitsversorgung*	
Aus Angst vor einer Fehlbehandlung eine Testung auf COVID‑19 vermieden	40 (2,7 %)
Aus Angst vor Diskriminierung eine Testung auf COVID‑19 vermieden	70 (4,7 %)
Würde eine Testung auf COVID‑19 aus Angst vor einer Fehlbehandlung vermeiden	127 (8,4 %)
Würde eine Testung auf COVID‑19 aus Angst vor Diskriminierung vermeiden	211 (14,0 %)
Haben im Zuge einer Testung auf COVID‑19 bereits Fehlbehandlungen erlebt	30 (2,0 %)
Haben im Zuge einer Testung auf COVID‑19 bereits Diskriminierung erlebt	31 (2,1 %)

^a^ Die Prozentangaben beziehen sich auf die Anzahl an Personen, die die entsprechende Behandlung in Anspruch genommen haben, nicht auf die gesamte Stichprobe

### Zugang zu trans-spezifischen Behandlungen

**Figure 1:**
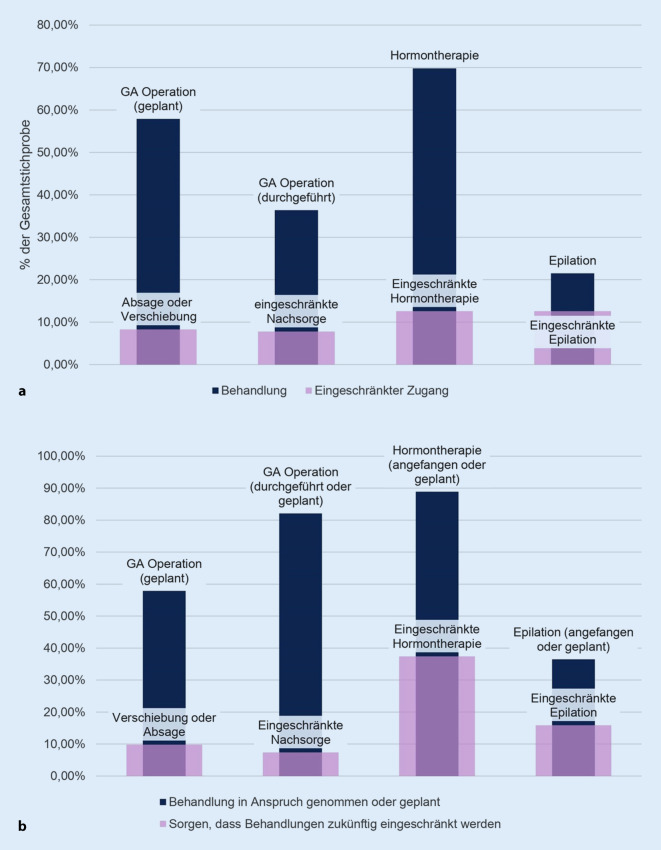

Von den 1101 TN (73,1 %), die zum Befragungszeitpunkt geschlechtsangleichende Maßnahmen in Anspruch nahmen, berichteten 490 (36,4 %) einen eingeschränkten Zugang zu mindestens einer dieser Maßnahmen (Abb. 1a). Am stärksten wurden die Epilationsbehandlungen eingeschränkt (59,9 %), gefolgt von der Nachsorge nach einer geschlechtsangleichenden Operation (21,9 %) und der Hormontherapie (18,1 %; Tab. 3). Weiterhin berichteten 771 TN (51,2 %) von den 1392 TN (92,4 %), die eine geschlechtsangleichende Maßnahme geplant oder in Anspruch genommen hatten, dass sie über zukünftige Einschränkungen aufgrund der COVID‑19-Pandemie besorgt waren (Abb. 1b). Weitere Informationen finden sich in Tab. 3.

**Table 3:** 

Angaben der Teilnehmenden	Absoluter und relativer Anteil
*Habe eingeschränkten Zugang zu mindestens einer Behandlung erlebt*	490 (36,4 %)
Eingeschränkter Zugang zu Hormonbehandlungen	190 (18,1 %)
Ich bekomme kein Rezept für meine Hormonpräparate	25 (13,2 %)
Ich bekomme keinen Termin bei eine_r_m Endokrinolog:in	79 (41,6 %)
Ein bereits vereinbarter Termin wurde ersatzlos abgesagt	35 (18,4 %)
Ein bereits vereinbarter Termin wurde verlegt	25 (13,2 %)
Ich traue mich nicht eine Arztpraxis oder ein Krankenhaus aufzusuchen	59 (31,1 %)
Meine Hormonpräparate können nicht oder nur eingeschränkt geliefert werden	70 (36,8 %)
Andere	53 (27,9 %)
*Weitere Angaben zu Einschränkungen bei der Behandlung*	
Sorgen, dass Hormonbehandlung in der Zukunft wegen COVID‑19 eingeschränkt wird	564 (42,8 %)
Der Zugang zu Epilationsbehandlungen ist aktuell eingeschränkt	190 (59,9 %)
Sorgen, dass Epilationsbehandlungen in der Zukunft wegen COVID‑19 eingeschränkt sein werden	239 (45,5 %)
*Operationstermin abgesagt oder verlegt*	
Ja	125 (14,5 %)
Noch nicht, aber ich erwarte es	147 (17,1 %)
*Probleme mit der Nachsorge einer vor Kurzem durchgeführten OP*	
Ich bekomme keinen Termin zur Nachsorge	22 (4,1 %)
Ein bereits vereinbarter Termin wurde ersatzlos abgesagt	25 (4,7 %)
Ein bereits vereinbarter Termin wurde verlegt	24 (4,5 %)
Komplikationen (z. B. Nachblutungen) wurden nicht behandelt	8 (1,5 %)
Ich traue mich nicht, eine Arztpraxis oder ein Krankenhaus aufzusuchen	11 (2,1 %)
Andere	27 (5,0 %)
*Weitere Angaben zu Einschränkungen bei der Nachsorge*	
Sorgen, dass die Nachsorge in der Zukunft wegen COVID‑19 eingeschränkt wird	111 (21,2 %)
*Eingeschränkter Zugang zu*	
Medizinischem Material, das nach einer Operation wichtig ist (z. B. Vaginaldilatatoren, Brustkompressen)	35 (2,3 %)
Weiteren Maßnahmen (z. B. Binder, Packing-Material)	160 (10,6 %)
Nichtmedizinischem Hilfsmaterial (z. B. Make-up, Rasierklingen, Perücken)	140 (9,3 %)
*Eingeschränkter Zugang zu Trans-Beratungsstellen*	593 (39,3 %)
Zugang zu den Angeboten der Trans-Beratung auf anderen Wegen möglich	406 (54,8 %)
*Mitglied einer Selbsthilfegruppe*	297 (19,7 %)
Zugang zu Selbsthilfegruppen eingeschränkt	280 (94,6 %)

Das logistische Regressionsmodell zur Untersuchung der Prädiktoren für vermehrte Einschränkungen im Zugang zu geschlechtsangleichenden Behandlungen erlangte statistische Signifikanz, χ^2^ = 104,126, *p* < 0,001 (Tab. 4). TN, denen bei der Geburt ein männliches Geschlecht zugewiesen wurde, haben demnach ein höheres Risiko, einen erschwerten Zugang zur Trans-Gesundheitsversorgung zu erleben (OR = 3,062). Ebenso haben Personen mit niedrigerem Einkommen ein höheres Risiko für Zugangseinschränkungen (OR = 1,139).

**Table 4:** 

Prädiktoren	*B* ± *SE*	*p*	OR [95 % KI]
Konstante	−1,541 ± 0,282	*<* *0,001*	0,214
Binäre geschlechtliche Selbstbezeichnung	0,202 ± 0,177	0,252	1,224 [0,866; 1,730]
Männliches Geschlecht zugewiesen bei der Geburt	1,119 ± 0,282	*<* *0,001*	3,062 [2,381; 3,937]
Einkommen	0,130 ± 0,46	*0,005*	1,139 [1,041; 1,246]
Zugehörig zu einer religiösen Minderheit	0,326 ± 0,180	0,070	1,385 [0,974; 1,969]
Zugehörig zu einer sexuellen Minderheit	−0,170 ± 0,193	0,380	0,844 [0,587; 1,233]
Selbstbezeichnung als Person mit Behinderung	0,102 ± 0,149	0,497	1,107 [0,826; 1,484]
Mindestens eine körperliche Erkrankung	0,015 ± 0,131	0,910	1,015 [0,768; 1,311]

*B* Regressionsgewicht, *SE* Standardfehler, *p* P-Wert, *OR* Odds Ratio/Chancenverhältnis; Cox-Snell *R*^*2*^ = 0,082, Nagelkerke *R*^*2*^ = 0,113, χ^2^(7) = 104,126, *p* < 0,001

### Psychische Belastung

Der Mittelwert des GSI für psychische Belastung lag für die Gesamtstichprobe bei 16,57 (SD = 12,96). Das Regressionsmodell zur Untersuchung der Prädiktoren für einen höheren GSI erlangte statistische Signifikanz, *F* (8, 1255) = 25,54, *p* < 0,001 (Tab. 5). Ein bei der Geburt zugewiesenes männliches Geschlecht war signifikant mit niedrigeren GSI-Werten assoziiert (*B* = −2,503, *p* = 0,001). Ein niedrigeres Einkommen (*B* = 2,074, *p* < 0,001), die Zugehörigkeit zu einer sexuellen Minderheit (*B* = 3,722, *p* < 0,001), die Selbstbezeichnung als Person mit Behinderung (*B* = 3,870, *p* < 0,001) und das Vorhandensein von mindestens einer körperlichen Erkrankung (*B* = 3,242, *p* < 0,001) waren signifikant mit höheren GSI-Werten assoziiert.

**Table 5:** 

Prädiktoren	*B* *±* *SE*	95 % KI	ß	*p*
Konstante	4,098 ± 1,550	[1,056; 7,140]	–	–
Eingeschränkter Zugang	0,235 ± 0,732	[−1,201; 1,671]	*0,009*	0,748
Binäre geschlechtliche Selbstbezeichnung	−1,962 ± 0,820	[−3,587; −0,337]	−0,064	0,018
Männliches Geschlecht zugewiesen bei der Geburt	−2,503 ± 0,727	[−3,929; −1,076]	−0,095	0,001
Einkommen	2,074 ± 0,248	[1,586; 2,561]	0,224	< 0,001
Zugehörig zu einer religiösen Minderheit	1,312 ± 0,978	[−0,607; 3,230]	*0,036*	0,180
Zugehörig zu einer sexuellen Minderheit	3,722 ± 1,057	[1,648; 5,796]	0,094	< 0,001
Selbstbezeichnung als Person mit Behinderung	3,870 ± 0,811	[2,280; 5,460]	0,129	< 0,001
Mindestens eine körperliche Erkrankung	3,242 ± 0,705	[1,858; 4,626]	0,124	< 0,001

*B* Regressionsgewicht, *SE* Standardfehler, 95 % *KI* Konfidenzintervall zum Niveau 95 %; *ß* = Regressionskoeffizient, *p* P-Wert; *R*^*2*^ = 0,149, Adj. *R*^*2*^ = 0,155, *F* (8, 1255) = 25,54, *p* < 0,001

## Diskussion

Ausgangspunkt dieser in Deutschland, der Schweiz und Österreich durchgeführten Studie war die Frage, welche Auswirkungen die COVID‑19-Pandemie auf die Gesundheit und die Gesundheitsversorgung von trans Personen hat. Hierbei fokussierten wir auf die unterschiedlichen Erfahrungen von trans Personen, die Maßnahmen der Trans-Gesundheitsversorgung in Anspruch genommen haben und nehmen.

Wie auch andere Studien fand die vorliegende Arbeit, dass der Zugang zu geschlechtsangleichenden Behandlungen (z. B. Hormontherapie, Epilation, geschlechtsangleichende Operationen) für trans Personen während der COVID‑19-Pandemie eingeschränkt war [11–14]. Der Vergleich zu anderen Ländern zeigt hier ein differenzierteres Bild, wobei TN aus dem deutschsprachigen Raum spezifisch von bestimmten Einschränkungen betroffen zu sein scheinen [11]. So berichten in den USA trans Personen fast doppelt so häufig von einem erschwerten Zugang zur Hormontherapie. Demgegenüber wurden geschlechtsangleichende Operationen in Deutschland öfter verzögert oder abgesagt [12]. Das Erleben von Einschränkungen im Zugang zur medizinischen Versorgung (v. a. erschwerter Zugang zur Hormontherapie und Verzögerung oder Absage geschlechtsangleichender Operationen) wurde von zwei Merkmalen beeinflusst. Zum einen haben TN, denen bei der Geburt ein männliches Geschlecht zugewiesen wurde, ein höheres Risiko, mindestens eine Einschränkung zu erleben. Eine mögliche Erklärung hierfür könnte sein, dass diese Epilationsbehandlungen in Anspruch nehmen, die aufgrund der Kontaktbeschränkungen bei körpernahen Dienstleistungen nahezu vollständig ausgesetzt wurden. Darüber hinaus kann diskutiert werden, ob trans Personen, denen bei Geburt ein männliches Geschlecht zugewiesen wurde, häufiger Diskriminierungen erleben, was diesen Zusammenhang ebenfalls vermittelt haben könnte [36]. Außerdem war für TN mit einem niedrigen Einkommen das Risiko erhöht, Einschränkungen im Zugang zu geschlechtsangleichenden Maßnahmen aufgrund der COVID‑19-Pandemie zu erleben. Dieses Ergebnis stimmt mit Befunden aus weiteren Studien überein, die zeigen, dass Personen mit niedrigem sozioökonomischen Status während der COVID‑19-Pandemie finanziell und gesundheitlich benachteiligt wurden [37, 38]. Einschränkend ist anzumerken, dass die Effekte beider Prädiktoren klein waren [39].

Einschränkungen im Zugang zu geschlechtsangleichenden Maßnahmen können negative gesundheitliche Folgen für trans Personen haben [11–14]. Die Verschiebung einer geschlechtsangleichenden Operation oder auch die Unterbrechung bzw. Verschiebungen einer Hormontherapie kann die Zunahme von klinisch relevanter Depressivität und Ängstlichkeit bis hin zu manifesten Suizidgedanken bedingen [11]. Außerdem bedarf die medizinische Transition im Sinne einer professionellen Gesundheitsversorgung eines regelmäßigen Monitorings (z. B. benötigt eine Hormontherapie regelmäßige Kontrollen des Hormonstatus sowie möglicher Nebenwirkungen und ggf. Dosisanpassung; [40, 41]). Wenn Termine bei zuständigen Fachärzt:innen pandemiebedingt verschoben oder abgesagt werden mussten, kann dies zum Beispiel das Risiko steigern, mögliche sekundäre somatische Erkrankungen einer Hormonersatztherapie nicht rechtzeitig zu erkennen. Die unzureichende Nachsorge nach einer Operation geht darüber hinaus mit einem erhöhten Risiko für Wundheilungsstörungen oder anderen postoperativen Komplikationen einher [13]. Ein eingeschränkter Zugang zu Epilationsbehandlungen kann den Transitionsprozess ebenfalls verlängern, die Geschlechtsdysphorie aufrecht halten und das Risiko für Diskriminierungserfahrungen erhöhen [17]. Das Ausmaß und die potenziellen Auswirkungen der zusätzlichen, durch die COVID‑19-Pandemie bedingten Einschränkungen im Zugang zur Trans-Gesundheitsversorgung sollten vor dem Hintergrund der dargestellten Vulnerabilität von trans Personen als bedeutsames Risiko für die psychische und physische Gesundheit betrachten werden [20, 21, 23, 28]. Hinzu kommt, dass die TN der vorliegenden Studie angaben, im Vergleich zur europäischen Allgemeinbevölkerung öfter an akuten oder chronischen Erkrankungen zu leiden [42]. Einige dieser chronischen Erkrankungen (z. B. Lungenerkrankungen) stellen ein erhöhtes Risiko für einen schweren Verlauf einer COVID‑19-Erkrankung dar [43]. Außerdem erleben trans Personen öfter als die Allgemeinbevölkerung in verschiedenen Lebensbereichen Diskriminierungen [20, 21]. In diesem Zusammenhang berichten die TN der vorliegenden Studie ebenfalls über Diskriminierungen und Fehlbehandlungen während der Testungen auf SARS-CoV‑2. Bis zu 15 % gaben an, eine Testung aus Angst vor Diskriminierung und Fehlbehandlung zu vermeiden. Neben den genannten Einflüssen von Zugangseinschränkungen zur medizinischen Versorgung war eine höhere psychische Belastung außerdem mit einem niedrigeren Einkommen, der Zugehörigkeit zu einer sexuellen Minderheit, der Selbstbezeichnung als Mensch mit Behinderung sowie dem Vorhandensein mindestens einer körperlichen Erkrankung assoziiert. Verschärft wurden die genannten Probleme von trans Personen während der COVID‑19-Pandemie zuletzt durch einen erschwerten Zugang zu Communityressourcen, wie zum Beispiel Unterstützung durch Selbsthilfegruppen und Trans-Beratungsstellen, sowie zu psychiatrischer und/oder psychotherapeutischer Behandlung [12].

Die vorliegende Studie verdeutlicht, dass trans Personen während der COVID‑19-Pandemie vielfältige Einschränkungen im Zugang sowohl zur Trans- als auch zur allgemeinen Gesundheitsversorgung erlebt haben. Für trans Personen kann dies eine Unterbrechung oder Verzögerung ihrer Behandlungen bedeuten und somit eine Verschlechterung der Geschlechtsdysphorie, der psychischen Gesundheit und der Lebensqualität bedingen [44, 45]. Hinzu kommt, dass trans Menschen bereits vor der COVID‑19-Pandemie einen erschwerten Zugang zum Gesundheitssystem erlebten [27, 30].

Obwohl viele Bereiche des Gesundheitssystems mit mangelnden Ressourcen und eingeschränkten Behandlungsmöglichkeiten wegen der COVID‑19-Pandemie zu kämpfen haben, sollte berücksichtigt werden, dass diskriminierte und marginalisierte Gruppen stärker von dieser Ressourcenknappheit betroffen sein können als die Allgemeinbevölkerung [37, 38, 46]. Basierend auf den Daten der vorliegenden Studie kann dies auch für trans Personen angenommen werden. Die pandemiebedingt erlebten Einschränkungen addieren sich zu einer bereits vorhandenen sozialen und medizinischen Vulnerabilität und verschärfen somit das Risiko von trans Personen, sowohl psychisch als auch physisch schwerwiegende gesundheitliche Probleme zu entwickeln.

## Limitationen und Ausblick

Die Erhebungsmethode via Online-Befragung begrenzt die Aussagekraft der analysierten Daten, da ausschließlich Personen mit einem internetfähigen Endgerät und hinreichenden technischen Kenntnissen an dem Survey teilnehmen konnten. So müssen wir annehmen, dass dieses Befragungsformat die Stichprobe in Richtung jüngerer, höher gebildeter TN verzerrt hat. Auch durch die Verbreitung der Studie über soziale Medien und Mailinglisten wird es wahrscheinlicher, dass Personen mit einer stärkeren Nutzung sozialer Medien eher an der Studie teilgenommen haben. Diese Einschränkungen gelten grundsätzlich für webbasierte Studien. Eine weitere wichtige Einschränkung der vorliegenden Studie ist das Fehlen einer Kontrollgruppe. Die erhobenen Daten können lediglich mit vorherigen Studien verglichen werden, die sich in Design und Methodik unterscheiden können. Weiterhin ist die fehlende Beteiligung der Praxispartner:innen an allen Schritten dieser Untersuchung eine Limitation der Studie. Aufgrund der rapiden Entwicklung der Pandemie haben die Autor:innen Schnelligkeit vor Partizipation priorisiert, um die erhobenen Daten so zeitnah wie möglich veröffentlichen zu können [47]. Zukünftige Forschung sollte diese Einschränkungen adressieren, z. B. indem die Einschränkungen beim Zugang zur Trans-Gesundheitsversorgung aufgrund externer Ereignisse longitudinal untersucht werden. Weithin sollten auch die langfristigen Folgen der beschriebenen Einschränkungen im Zugang zur Trans-Gesundheitsversorgung auf die physische und psychische Gesundheit von trans Personen erfasst werden.

## Schlussfolgerung

Für die Situation von trans Personen in Deutschland, der Schweiz und Österreich fand die vorliegende Studie vielfach Einschränkungen im Zugang zur Gesundheitsversorgung durch die COVID‑19-Pandemie. Der Zugang sowohl zu transitionsunterstützenden Behandlungen als auch zur allgemeinen Gesundheitsversorgung wurde bei gleichzeitig zunehmender Isolation von der Trans-Community eingeschränkt. Vor dem Hintergrund der bereits vor der COVID‑19-Pandemie dokumentierten Vulnerabilitäten von trans Personen zeigen die empirisch erfassten Einschränkungen, dass sich die gesundheitlichen Risiken von trans Personen im Rahmen der COVID‑19-Pandemie maßgeblich verschärft haben.

## Footnotes

1 Der Begriff Minoritätenstress beschreibt spezifische Stressoren, die (u. a. sexuelle oder geschlechtliche) Minderheiten aufgrund von Stigmatisierung und Marginalisierung durch die Gesamtgesellschaft erleben [22].

2 Der Begriff Transition umfasst alle sozialen, juristischen und medizinischen Schritte, die im Prozess der Geschlechtsangleichung durchgeführt werden können [17].

3 Der Begriff cis beschriebt alle Personen, deren geschlechtliche Selbstbezeichnung mit dem bei der Geburt zugewiesenen Geschlecht übereinstimmt (cis: lateinisch für „diesseits“, trans: „jenseits“).

4 Der vorliegende Beitrag nutzt im Sinne einer geschlechtergerechten Schreibweise einen Doppelpunkt [24]. Es ist den Autor:innen bewusst, dass diese sowie weitere geschlechtergerechte Schreibweisen in Teilen kritisch diskutiert werden [25, 26].

5 Lesbian, Gay, Bi, Trans, Queer, + weitere geschlechtsbezogene Identitäten und sexuelle Orientierungen.
